# Influence of Selected Warm Mix Asphalt Additives on Cracking Susceptibility of Asphalt Mixtures

**DOI:** 10.3390/ma13010202

**Published:** 2020-01-03

**Authors:** Marcin Stienss, Cezary Szydlowski

**Affiliations:** Faculty of Civil and Environmental Engineering, Gdańsk University of Technology, 80-233 Gdańsk, Poland; cezary.szydlowski@pg.edu.pl

**Keywords:** warm mix asphalt, cracking susceptibility, semi-circular bending test, TSRST, low temperature properties

## Abstract

Warm mix asphalt (WMA) has been widely accepted as a future asphalt paving technology. Besides clear advantages, there are still some concerns regarding durability and long-term performance of pavements made with this type of asphalt mixtures. One of the most important issues is low temperature behaviour of WMA because certain additives used for temperature reduction can affect bitumen properties. This paper presents the evaluation of low-temperature properties of laboratory-produced asphalt concrete for wearing course with selected WMA additives. One type of bitumen with paving grade 50/70 and five WMA additives of different nature (organic, surface tension reducer and combination of both) were used in this study. The production and compaction temperature of mixtures containing WMA additives was 25 °C lower in comparison with the temperature of the reference mix. To assess the susceptibility of WMA to low-temperature cracking, Semi-Circular Bending (SCB) and Thermal Stress Restrained Specimen Test (TSRST) were used. Supplementary rating was made by analysing Bending Beam Rheometer (BBR) test results of asphalt binders.

## 1. Introduction

### 1.1. Background

Warm mix asphalt technology is continuously gaining bigger share in total production volume of asphalt mixtures. This is due to the fact that it offers considerably advantages over traditional hot mix asphalt, both in terms of technical aspects as well as in environmental issues [1,2,3], which is indispensable in today’s economy. There are several different technologies of reducing asphalt mixture working temperatures. These can be divided into three major groups: organic additives, chemical additives, and foaming technologies based on zeolite minerals or direct water injection into the binder stream with the use of plant-based systems [4]. Techniques including a combination of different processes are also being considered [5,6]. According to recent surveys conducted in USA, warm asphalt mixtures gained almost a 41% share in the total volume of all asphalt mixtures produced in 2018. Furthermore, after a period of 2009–2014 when plant-based foaming processes formed the majority of WMA production at the level of 85%–95%, it is now observed that additive technologies regained part of the market and constituted 34% of total WMA production in 2018 [7]. Thus, research efforts concerning warm mix asphalt additives are considered to be still when actual and new additives are being proposed [8,9,10,11,12,13] or combined with other substances [14,15,16] to achieve a higher degree of asphalt mixture sustainability.

There are many studies concerning fundamental properties of warm mix asphalt mixtures produced with additives, especially in the area of moisture susceptibility and resistance to permanent deformation [17,18,19,20,21,22] because these are the most significant issues when producing and laying such mixtures during which the temperature is reduced and, thus, there is a risk of not obtaining a proper degree of aggregate coating and compaction. Studies concentrated on low-temperature cracking behavior with the use of dedicated tests were less frequent. Yoo et al. [23] used three points bending geometry and rectangular notched beams to assess fracture toughness K_IC_ of the WMA mixture with a wax type additive. No negative effects were observed in terms of fracture resistance and WMA mixture performed equivalently or even better than the reference hot mix asphalt (HMA) mixture. Results of fracture tests that were conducted by Bernier et al. [24] for traditional hot asphalt mixture and two warm asphalt mixtures based on wax type additive and foaming technology indicated that there were no significant differences in the results of fracture toughness and energy obtained with a semi-circular bending test (SCB) and disk-shaped compact tension test (DCT), even though the pavement field section with a wax-based additive experienced the highest degree of cracking in comparison with two other sections. Das et al. [25,26] studied the effect of adding relatively high percentages of commercially available waxes (4%) on binder and asphalt mixture low-temperature performance. Although the stiffening effect observed in the binder was significant, the negative effect in the asphalt mixture expressed as changes of the TSRST fracture temperature and Indirect Tension IDT creep compliance were recognized as minor. Hill et al. [27] used three methods for evaluating WMA low temperature performance: DCT, IDT, creep compliance, and Acoustic Emission (AE) and concluded that the behavior of WMA mixtures with additives depends on the type of the used additive. While organic and foaming additives reduced fracture energy, chemical additives improved it. TSRST tests conducted by Medeiros et al. [28] showed that the fracture and transition temperature of WMA mixtures with certain additives could be higher in comparison with control HMA and this can be a sign of reduced performance at low temperatures. According to Hajj et al. [29], slightly lower TSRST fracture temperature of the warm asphalt mixture, which was tested in his research, could be a result of lower short-term ageing temperature. A negative impact of wax type WMA additives on the asphalt mixture stress intensity factor was observed by Hasan et al. [30]. Lower production temperature of warm mix asphalt could be very beneficial for asphalt mixtures that contain high percentages of crumb rubber [31] and/or recycled asphalt pavement because unfavourable increases of mixing and compaction temperature could be mitigated by the incorporation of WMA additives. Singh et al. [32] conducted experiments with mixtures containing up to 40% percent of reclaimed asphalt pavement combined with two WMA additives that are wax-based and chemical-based. Cracking susceptibility was evaluated by means of the SCB test. In overall, it was suggested that WMA additives may contribute to the degradation of intermediate fracture characteristics, but the WMA wax type additive showed better results than the chemical type. Among others, work in this area was also done by Razmi et al. [33] and Cao et al. [34]. To summarize, previous research results were, in some cases, inconclusive and sometimes field observations were inconsistent with laboratory results [35,36].

### 1.2. Objectives

The aim of the research described in this paper was to evaluate the influence of selected warm mix asphalt additives on low temperature behavior of asphalt mixtures expressed in terms of tensile strength (uniaxial tension stress test) and fracture properties (fracture toughness and fracture energy). These parameters were also discussed and confronted with the results of binder tests.

## 2. Materials and Methods

### 2.1. Materials

#### 2.1.1. Bitumen

For this study, neat bitumen 50/70 produced in polish refinery was selected. This type of bitumen is widely used in Poland for wearing courses of roads with light and medium traffic (from 0.03 × 10^6^ to 7.3 × 10^6^ of 100 kN standard axle loads, which correspond to 0.07 × 10^6^ and 17.8 × 10^6^ of 80 kN standard axle loads).

#### 2.1.2. Warm Mix Asphalt Additives

Five different warm mix additives were used in this study. The range of additives was selected in a way to represent most of the types of additives available on the market. The percentage of dosage of each additive to neat bitumen was established on the basis of literature studies and producer’s guidelines. Designation, short description, form, and dosage of each additive are shown in Table 1.

The first phase of the sample preparation process was adding warm mix asphalt additives to the neat 50/70 bitumen, which was done a day before actual production and compaction of asphalt mixture samples. This procedure involved preheating of a needed amount of bitumen to the temperature of 135 °C and adding previously weighed amounts of each additive to the respective canisters and mixing with portable high-shear mixer (rotor-stator type) for 2–3 min. After that, closed canisters with bitumen mixed with WMA additives were stored for 1 hour at the temperature of 135 °C. Afterwards, before using in the mixture, bitumen was additionally mixed manually for 15 min. Neat bitumen 50/70 for reference samples was stored for 1 hour before production and compaction at the temperature of 160 °C. The same temperatures were used during the mixing and compaction process, which means 25 °C reduction for warm mix asphalt mixtures with additives in comparison with a reference mixture produced with neat 50/70 bitumen. For every type of the asphalt mixture test, a specific amount of binder was blended with the WMA additive, and was used only once. There was no additional cooling and heating process of mixed cans.

Table 2 summarizes properties of neat bitumen 50/70 and bitumen with each additive respectively. s 1 and 2 present the results of the BBR test at −12 °C. These results are presented to show the influence of tested WMA additives on creep characteristics obtained at a low Performance Grade (PG) temperature of the 50/70 binder.

**Figure 1:**
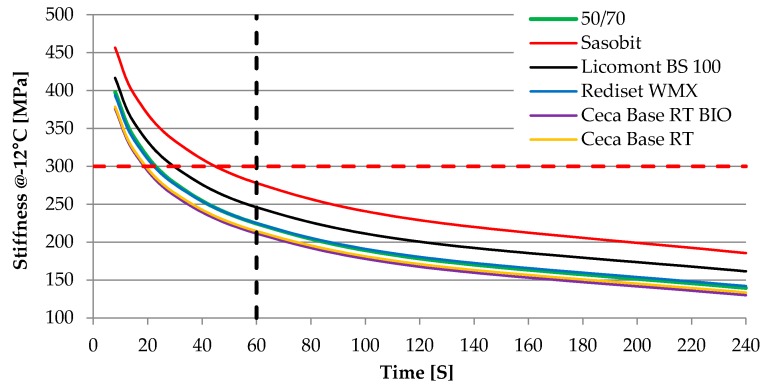
Stiffness obtained at –12 °C from the BBR test.

**Figure 2:**
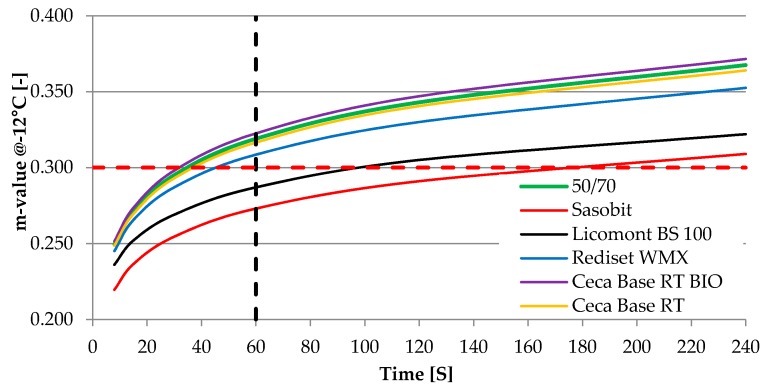
m-value obtained at –12 °C from the BBR test.

As can be seen in Figure 1; Figure 2, two wax-based WMA additives have a significant influence on properties of neat 50/70 binder, both in terms of the stiffness increase and the m-value reduction. The change of the m-value shifts PG-grade in these two cases to the next level, from 64−22 to 70−16. Changes in PG grade strictly correspond with changes of basic bitumen properties – penetration and softening point. For other WMA additives that incorporate surfactant-based molecules and act as adhesion promoters, such an influence is minor and does not change PG classification. For two liquid WMA additives, results indicate that the binder becomes slightly softer.

#### 2.1.3. Asphalt Mixture

**Figure 3:**
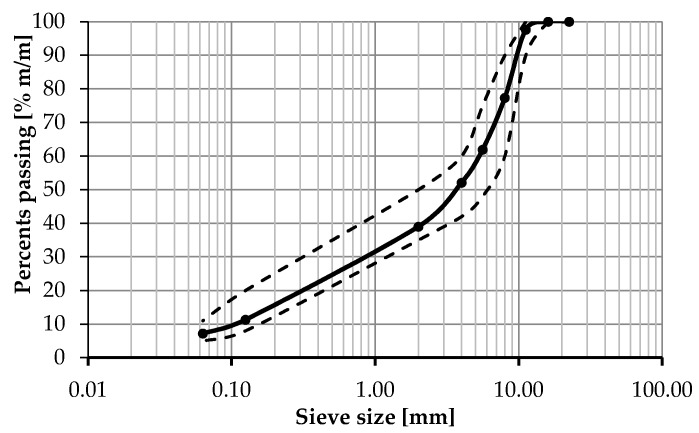
Grading curve of the used asphalt concrete mix and grading envelope according to the technical guidelines WT-2:2014 [38].

In this study, asphalt concrete AC 11S for wearing course for medium traffic KR3÷4 (from 0.5 × 10^6^ to 7.3 × 10^6^ of 100 kN standard axle loads, which correspond to 1.2 × 10^6^ and 17.8 × 10^6^ of 80 kN standard axle loads) was used, which was designated in accordance with standard EN 13108-1 [37] and designed in accordance with Polish technical guidelines WT-2:2014 [38]. The mineral mixture was composed of crushed gneiss/granite and mineral limestone filler. The same mineral aggregate was used for all variants of tested bitumen with different warm mix asphalt additives. Asphalt binder content was based on the requirements of technical guidelines [38] that set minimum binder content in correspondence with the pavement course type and traffic level and also take into account the correction factor calculated on the basis of mineral mixture density. The anti-stripping agent was not used so as not to influence tests results. Table 3 presents basic properties of the used asphalt concrete mixture. Figure 3 presents the grading curve and grading envelope.

#### 2.1.4. Samples Preparation

Asphalt mixtures were prepared with the use of the laboratory mixer in accordance with the EN 12697-35 standard [39]. Before mixing, the aggregate was heated up to 160 °C in case of the reference mixture with neat 50/70 bitumen and 135 °C in case of the mixtures with WMA additives. Mixing time was set to 5 min. Prior to compaction of specimens, asphalt mixtures were subjected to low-term ageing in accordance with the procedure given in the Appendix 2 of the WT-2:2014 [38].

Samples for a uniaxial tension test were compacted with the use of roller compactor according to standard EN 12697-33 [40] into rectangular slabs with dimensions of 305 × 305 mm and thickness of 80 mm. The amount of mixture placed in the mould before compaction was calculated in a way to obtain the final degree of compaction at the level of 99% of the Marshall specimen bulk density.

After compaction, the initial samples were stored for one day in room temperature and cut down to the desired shapes and dimensions of 40 mm × 40 mm × 160 mm. Three such specimens of each asphalt mixture were prepared. The specimens were cut from the internal volume of the initial slabs so as to obtain a minimum distance between edges of at least 20 mm and to discard side areas that could contain higher air voids.

Samples intended for the semi-circular bending test were compacted with the use of the gyratory compactor, with a diameter of 150 mm and a height of 105 mm. The compaction process was also set up to obtain 99% of Marshall density. For every type of asphalt mixture and test temperature, four specimens were prepared.

Samples were produced to obtain 99% of Marshall density to meet Polish requirements regarding functional testing of asphalt mixtures [38]. The compaction index and air voids content were determined for every sample after cutting. Only samples with a compaction index from 98.5% to 99.5% were allowed to be used for the TSRST and SCB test.

### 2.2. Tests Methods

#### 2.2.1. Uniaxial Tension Tests

Tensile strength of asphalt mixtures at low temperatures was evaluated by means of the uniaxial test method, which includes thermal stress restrained specimen (TSRST), according to standard EN 12697-46 [41] and with the use of TSRST–MULTI Multi-Station Thermal Asphalt System servo electric equipment (PAVETEST, Italy). During the TSRST test, the rectangular sample is subjected to a continuously decreasing temperature. The standard initial temperature *T*_0_ is set to 20 °C and the rate of cooling is constant and equal to 10 °C/h. Because the specimen is clamped to assure constant length throughout the test, thermal shrinkage is prohibited and, thus, cryogenic (thermal) stress builds up until the specimen fractures. At the breaking point, the stress reaches its maximum value and is defined as the failure stress σ_cry, failure_ and the temperature at the breaking point is defined as the failure temperature T_failure_. In low temperatures, the slope of the stress–temperature curve Δσ/ΔT is close to the straight line, which means a linear elastic characteristic of the tested asphalt mixture. Values obtained through the test are (1) progression of the cryogenic (thermal) stress over the temperature σ_cry_(T) and (2) failure stress σ_cry_ at the failure temperature T_failure_, which equals the tensile strength of the specimen at the failure temperature. The temperature at the tangent point T_g_ is defined by the intersection between the two tangents of the stress-temperature curve at the elastic section and at the stress relaxation section, which is assumed to be around the starting point of the test at a temperature of 20 °C.

**Figure 4:**
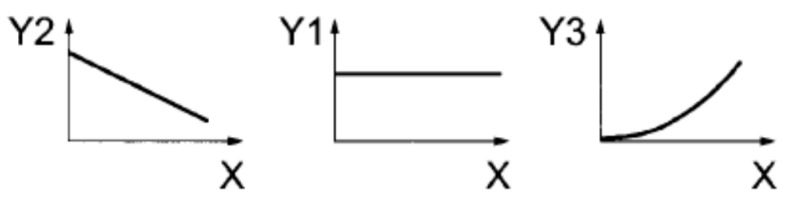
The TSRST test principle with restrained thermal stress, where: X—time, Y1—strain, Y2—temperature, and Y3—stress [41].

**Figure 5:**
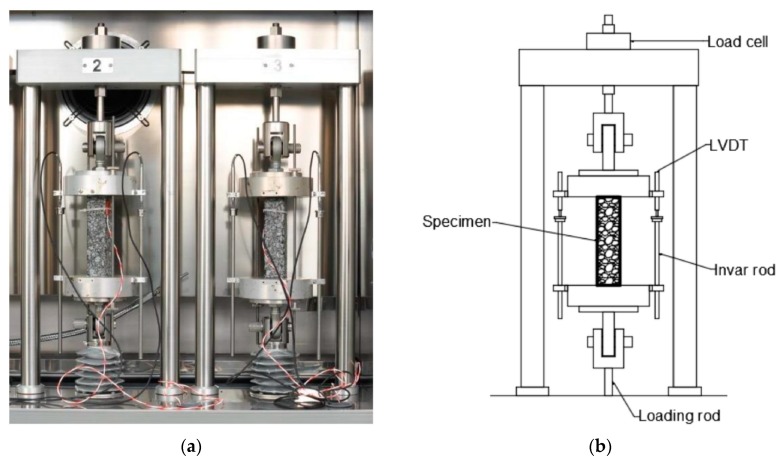
Thermal stress restrained specimen test (TSRST) setup: photograph of specimens during the test (**a**) and schematic view (**b**).

Figure 4 shows the principle of the TSRST test. Figure 5 presents setup of the used equipment.

**Figure 6:**
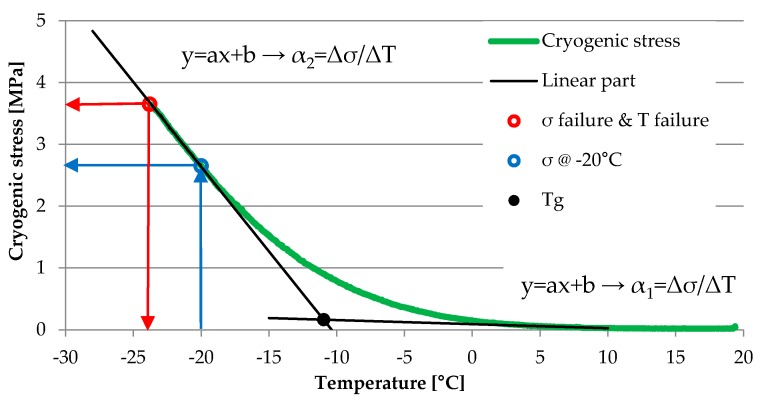
Graphical explanation of data assessment from TSRST.

The graphical explanation of data obtained from the TSRST test is shown in Figure 6.

At lower temperatures, the slope of the stress-temperature curve Δσ/ΔT becomes linear (constant), which means that the asphalt mixture behaves like an elastic material. The temperature at the tangent point (T_g_) is defined by the intersection between two tangents of the stress-temperature curve at the previously mentioned elastic zone and at the stress relaxation zone, which occurs around the start point of the test at the temperature of 20 °C.

#### 2.2.2. Semi-Circular Bending Test (SCB)

The Semi-Circular Bending Test (SCB) was conducted to evaluate fracture properties of tested asphalt mixtures. The test was based on the procedure described in standard EN 12697-44 [42] that was further modified on the basis of a literature review finding. In this method, the asphalt mixture resistance to fracture *K_IC_* is calculated using the following equation that takes into account maximum force recorded during three-point bending of the specimen.

(1) $$K_{I}=\sigma_{0}Y_{I}\sqrt{\pi a}$$

where: *a*—notch depth, *σ*_0_—test extreme stress, and *Y_I_* – normalized stress intensity factor due to type I fracture.

The extreme bending stress in the specimen was calculated using the following equation.

(2) $$\sigma_{0}=F/2rB$$

where: *F*—maximum test force, *r*—specimen radius, and *B*—specimen thickness.

The normalized stress intensity factor was calculated by the following equation.

(3) $$Y_{I}=4.782-1.219\left(a/r\right)+0.063\exp(7.045\left(a/r\right)$$

The critical value of the J-integral that characterizes the strain energy release rate during crack propagation was calculated from the relationship between the change in notch length that was cut in the bottom plane of the sample and the change of strain energy was measured to failure (pre-peak). The J-integral was calculated by the following equation.

(4) $$J_{C}=-\left(\frac{1}{B}\right)\frac{dU_{PRE-PEAK}}{da}$$

where: *U_PRE-PEAK_*—strain energy to failure of the specimen, *a*—depth of the notch, *B*—specimen thickness, and *dU_PRE-PEAK_/da*—change of strain energy with changing of notch depth.

In order to determine the change of strain energy with the change of notch depth, tests were carried out on specimens with different depths of the initial crack, which were 10 mm, 20 mm, and 30 mm. The rate of displacement was set to 1 mm/min. During the test, both specimen and loading frame were placed in a thermostatic chamber to maintain a constant temperature. In total, two different temperatures were used: −20 and +10 °C.

**Figure 7:**
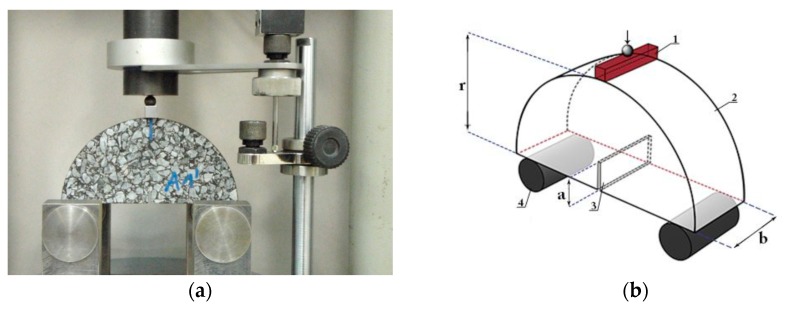
Semi-circular bending test (SCB) with the displacement strain rate setup: (**a**) photograph of the test setup. (**b**) Layout of the supports and loading point (1—loading rod, 2—sample, 3—notch, and 4—supporting rods).

The specimen during the SCB test and a scheme of the test are presented in Figure 7.

**Figure 8:**
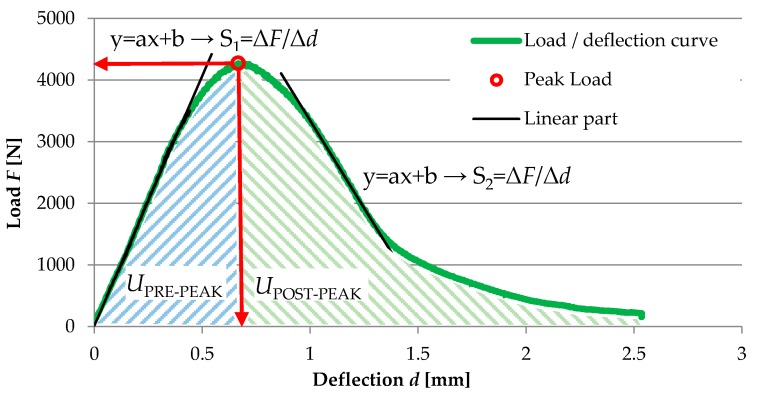
Graphical explanation of data assessment from the SCB test.

The graphical explanation of data obtained from the SCB test is shown in Figure 8.

## 3. Results and Discussion

### 3.1. Results Form the Thermal Stress Restrained Specimen Test (TSRST) and their Analysis

**Figure 9:**
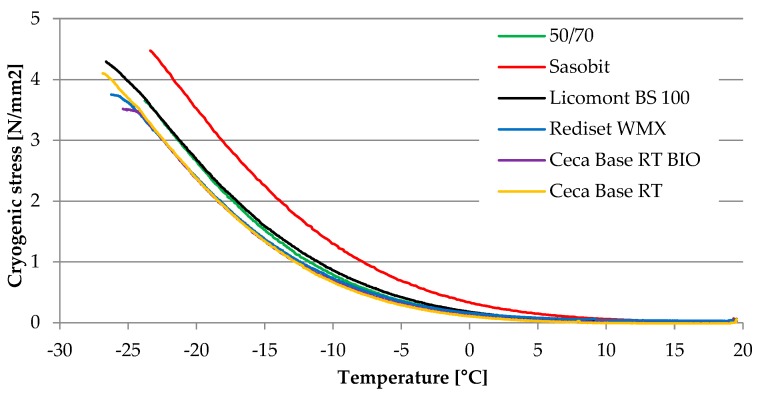
Results of thermal (cryogenic) stresses recorded during TSRST.

Selected results of cryogenic stresses obtained from the TSRST test for asphalt binder 50/70 with different warm mix additives are presented in Figure 9. Table 4 shows the analysis of TSRST test results. Besides the cryogenic stress and temperature values at failure (σ_cry, failure_ and T_failure_), slopes of curve tangents for elastic and stress relaxation zones were determined and discussed. For comparative purposes, cryogenic stress at the temperature of −20 °C (σ_cry, @−20°C_) was also analysed.

Results presented in Table 4 show that the general trend observed through the BBR test is strongly replicated by values of the TSRST test. For two wax-based WMA additives, the highest values of σ_cry, failure_ and σ_cry, @−20 °C_ were obtained while, for the rest of the additives, it can be assumed that stresses during fracture and at the temperature of −20 °C were mostly unchanged in comparison with a reference mixture produced with the neat 50/70 binder. A similar correlation could be formulated for failure temperature T_failure_ and T_g_ point that were highest for the Sasobit wax-based WMA additive. However, it should be noted that clear changes of bitumen properties, which shifted the binder with both wax-based WMA additives to the next level of the PG grade did not affect behaviour of asphalt mixtures to the same extent. Many values obtained through the TSRT test stay within a range of standard deviation. Moreover, results of the TSRST test should be evaluated with care since this test does not replicate field conditions in a strict way. For this research standard, the cooling rate of 10 °C/h was used while, in Poland, no cooling rate higher than 3 °C/h was recorded even in extreme conditions and the rate of cooling influences the strength reserve of the asphalt mixture [43].

### 3.2. Results of Fracture Toughness from the Semi-Circular Bending Test (SCB) and their Analysis

**Figure 10:**
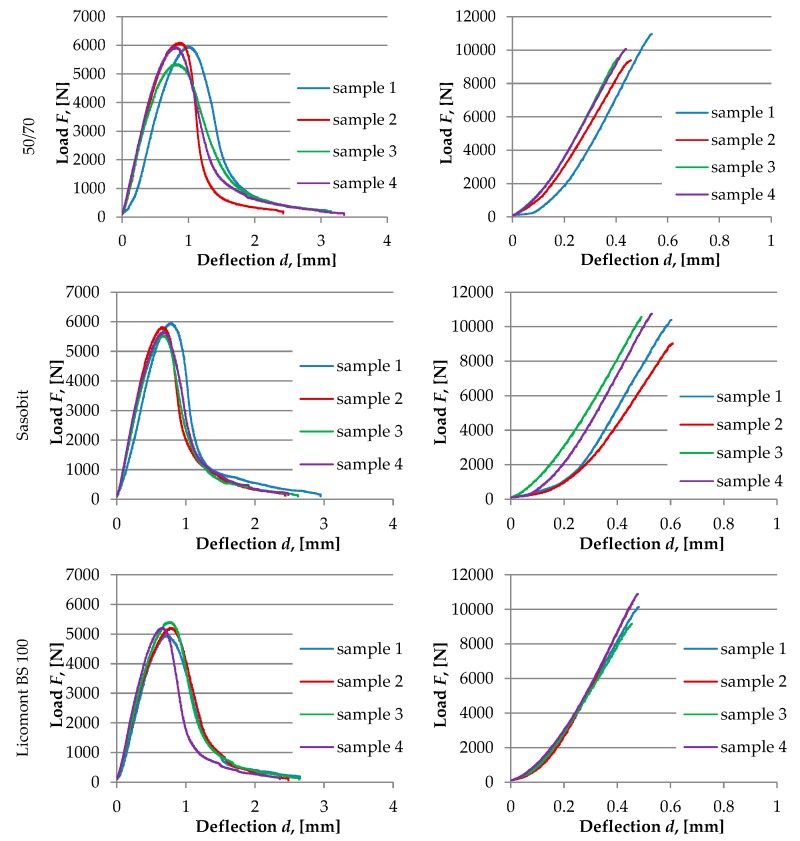

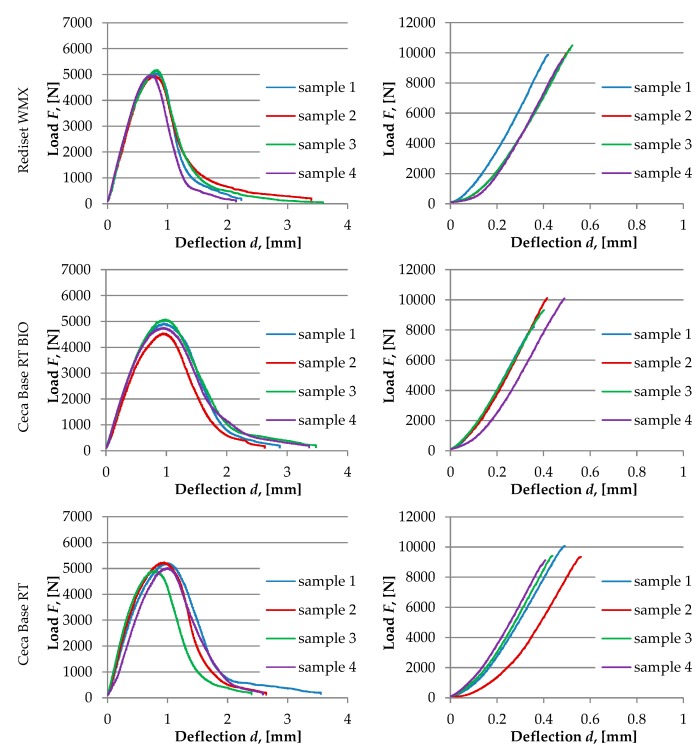
Selected results from the SCB test. Load/deflection curves for samples with a 10-mm notch. Left column: test temperature +10 °C. Right column: test temperature −20 °C.

**Figure 11:**
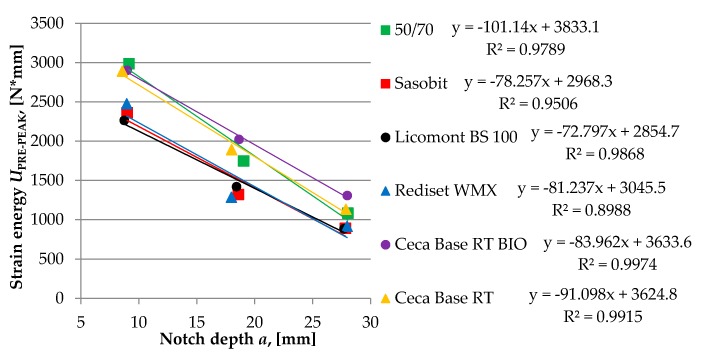
The graphical explanation *J*_C_ calculation from the SCB test at 10 °C.

**Figure 12:**
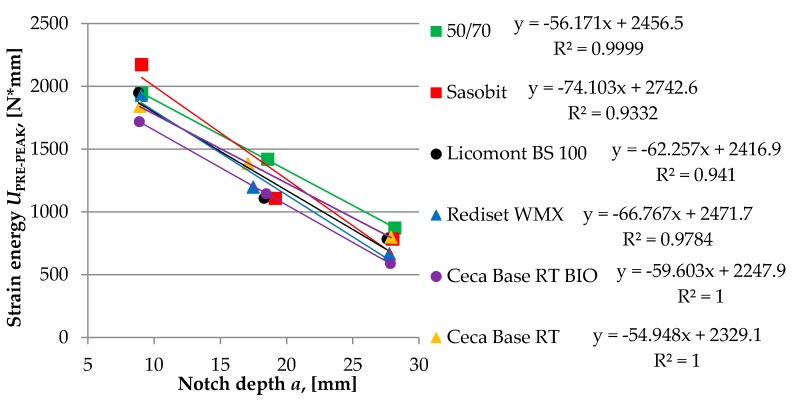
The graphical explanation of *J*_C_ calculation from the SCB test at −20 °C.

Test results for each type of asphalt mixture are shown in Figure 10, Figure 11 and Figure 12. Obtained numerical values are presented in Table 5 and Table 6.

Results of the SCB test conducted at an intermediate temperature of 10 °C are in accordance with previous results of the BBR test and TSRST test. Fracture energy of asphalt mixtures produced with two wax-based additives was lower than for other tested mixtures with different WMA additives, which may indicate that the risk of cracking could be higher if these mixtures would be subjected to real climatic conditions. Results obtained at the temperature of −20 °C are more inconclusive. In this case, a mixture with a wax-based Sasobit additive achieved the highest value of *J*_c_. This may be attributed to the fact that, at the temperature of −20 °C, the calculated fracture energy is based only on the pre-peak loading phase in contrast with the temperature of 10 °C for which both phases are taken into consideration.

## 4. Conclusions

The following paper presents the results of research concerning behavior or asphalt mixtures with selected WMA additives at low temperatures. The influence of additive type on basic bitumen properties, tensile strength (TSRST), and fracture toughness was evaluated and discussed. On the basis of obtained results and analysis, the following conclusions can be formulated.

1. Certain WMA additives, especially those belonging to the group of wax-based additives, can affect bitumen properties.
2. Additive that are based on surfactant-based molecules and incorporates a different type of temperature reduction mechanism based on adhesion promotion and reduced surface tension do not affect bitumen properties to such an extent.
3. Incorporation of wax-based WMA additives into bitumen can shift the PG grade to the next level. The initial PG grade of neat 50/70 bitumen was changed from 64-22 to 70-16 after adding two wax-based WMA additives. Thus, when using such additives, great care must be taken during the binder selection. In some cases, it would be likely advisable to initially select a binder with a lower PG grade, so as to obtain the desired PG level in terms of traffic requirements and climatic conditions after a WMA additive is incorporated. This issue requires further research.
4. Recorded changes of bitumen properties, which shifted the binder with some WMA additives to the next level of the PG grade did not reproduce in asphalt mixtures to the same extent. Values obtained through the TSRT test were mostly within a range of standard deviation. It should be remembered that the standard rate of cooling of 10 °C/h that was used in this study does not represent real cooling rates that may occur in real field conditions (which, in fact, are much lower) and this may attribute to the fact that, in this test, asphalt mixtures with wax-based additives showed similar performance to mixtures with other additives.
5. Results of the SCB test at the temperature of −20 °C were inconclusive and cannot be used for evaluating WMA additives. Results obtained for the temperature of 10° C were in accordance with the results of the BBR test and TSRST test.
6. This may be attributed to the fact that, at the temperature of −20 °C, during the SCB test with a constant rate of deformation, there is almost no post peak phase of loading. Destruction of the sample occurs almost immediately after the crack appears.
7. The conducted research and analysis of the test results do not disqualify any of the evaluated WMA additives in certain cases of pavement construction, traffic, and climatic conditions. They only show which asphalt mixture would be more prone to cracking when used in the field.
8. The correlation between cracking properties of WMA asphalt mixtures produced in laboratory conditions with samples extracted from the real pavements will be the subject of future studies. This is especially important since some of the additives that have shown a negative impact on the binder properties are continuously being used and pavements with such additives perform fairly well.

## Figures and Tables

**Table 1:** Description of warm mix asphalt additives used in the study.

Additive Designation	Chemical Composition	Type of Additive	Form
Sasobit	Aliphatic synthetic wax produced with the use of the Fisher–Tropsch method	Viscosity modifier	Granules
Licomont BS 100	Mixture of fatty acid derivatives	Viscosity modifier	Fine powder
Rediset WMX	Organic combined with adhesion promoter	Viscosity modifier and adhesion promoter of surface tension between the asphalt binder and aggregate	Pellets
Ceca Base Bio	Formulation of biodegradable and mostly bio-sourced surfactant-based molecules (ionic and non-ionic)	Adhesion promoter and reducer of the surface tension	Liquid
Ceca Base LQ	Formulation of surfactant-based molecules (ionic and non-ionic)	Adhesion promoter and reducer of the surface tension	Liquid

**Table 2:** Properties of bitumen 50/70 with warm mix asphalt additives.

Property	Designation of Bitumen or Additive					
	Neat Bitumen 50/70	Sasobit	Licomont BS100	Rediset WMX	Ceca Base BIO	Ceca Base LQ
Dosage rate of additive, % by wt. of asphalt binder	-	3%	3%	2%	0.35%	0.35%
Penetration at 25 °C, 0.1 mm, acc. to EN 1426	48.2	30.0	34.6	45.6	55.0	49.4
Softening point, °C, acc. to EN 1427	49.9	78.9	76.6	55.2	48.5	48.9
PG grade, acc. to AASHTO M 320	64−2	70−16	70−16	64−22	64−22	64−22
BBR S-critical temperature, °C	−12	−12	−12	−12	−12	−12
BBR m-critical temperature, °C	−12	−6	−6	−12	−12	−12

**Table 3:** Properties of the asphalt mixture.

Properties	Value
Maximum size of aggregate, mm	11
Binder content, wt.%	5.6
Air voids in Marshall samples (2 × 75 blows) [%]	3.1
Voids filled with bitumen VFB [%]	81.5
Voids in the mineral aggregate VMA [%]	16.7

**Table 4:** Results of the TSRST test.

Bitumen Type		σ_cry, failure_, [MPa]	T_failure_, [°C]	σ_cry, @-20°C_, [MPa]	α_2_, [N/mm^2^/°C]	α_1_, [N/mm^2^/°C]	T_g_, [°C]
50/70	mean value	3.926	−24.6	2.679	−0.286	−0.006	−11.2
	st. deviation	0.313	1.1	0.024	0.012	0.001	0.2
	CV, [%]	8.0	4.3	0.9	4.2	9.1	1.9
Sasobit	mean value	4.243	−23.3	3.382	−0.273	−0.014	−8.8
	st. deviation	0.224	0.1	0.151	0.007	0.003	0.2
	CV, [%]	5.3	0.4	4.5	2.4	17.6	2.0
Licomont BS 100	mean value	4.100	−25.3	2.736	−0.262	−0.009	−10.0
	st. deviation	0.273	1.4	0.072	0.006	0.000	0.6
	CV, [%]	6.7	5.4	2.6	2.3	0.0	5.7
Rediset WMX	mean value	3.872	−25.5	2.513	−0.267	−0.006	−11.2
	st. deviation	0.113	1.2	0.275	0.014	0.001	0.8
	CV, [%]	2.9	4.5	10.9	5.1	18.2	6.7
Ceca Base RT BIO	mean value	3.775	−25.7	2.409	−0.259	−0.007	−11.0
	st. deviation	0.242	0.5	0.053	0.002	0.001	0.2
	CV [%]	6.4	1.9	2.2	0.6	14.3	1.9
Ceca Base RT	mean value	4.073	−26.0	2.509	−0.262	−0.006	−10.9
	st. deviation	0.072	1.2	0.199	0.012	0.001	0.2
	CV [%]	1.8	4.5	7.9	4.5	18.2	2.1

**Table 5:** SCB test results at 10 °C.

Bitumen Type	Notch Depth [mm]	*F*_max_ [N]	*d*_Fmax_ [mm]	*K*_IC_ [N×mm^−3/2^]	*U*_PRE-PEAK_ [N×mm]	*U*_TOTAL_ [N×mm]	*S*_1_ [N/mm]	*S*_2_ [N/mm]	*J*_C_ [kJ/m^2^]
50/70	10	5837	0.88	19.8	2985	6188	9287	−12,572	1.98
	20	4272	0.71	21.3	1746	4170	7876	−6131	
	30	3052	0.6	19.9	1082	3092	6940	−4531	
Sasobit	10	5740	0.70	19.4	2360	4872	10,635	−15,564	1.53
	20	4302	0.53	21.1	1320	2966	10,149	−11,508	
	30	3398	0.50	22.1	890	2355	9077	−7575	
Licomont BS 100	10	5200	0.73	17.3	2265	4687	9495	−10,900	1.41
	20	3939	0.63	19.3	1422	3236	8834	−8098	
	30	3049	0.56	19.7	886	2436	7163	−4380	
Rediset WMX	10	5033	0.79	16.9	2476	4936	8256	−9422	1.59
	20	3546	0.61	17.1	1285	3310	7828	−6200	
	30	2835	0.54	18.5	915	2690	6611	−3527	
Ceca Base RT BIO	10	4828	0.94	16.3	2902	6473	6943	−5537	1.65
	20	4052	0.79	19.9	2023	4914	7126	−4908	
	30	3123	0.70	20.3	1308	3465	5838	−4560	
Ceca Base RT	10	5091	0.96	16.7	2892	6023	8184	−7514	1.77
	20	3711	0.83	17.9	1891	4338	6013	−4327	
	30	2850	0.63	18.5	1132	3288	6723	−3927	

**Table 6:** SCB test results at −20 °C.

Bitumen Type	Notch Depth [mm]	*F*_max_ [N]	*d*_Fmax_ [mm]	*K*_IC_ [N×mm^−3/2^]	*U*_PRE-PEAK_ [N×mm]	*S*_1_ [N/mm]	*J*_C_ [kJ/m^2^]
50/70	10	9988	0.47	33.7	1945	28,197	1.10
	20	7469	0.51	36.6	1419	24,895	
	30	5230	0.4	34.3	872	18,478	
Sasobit	10	10,187	0.59	34.4	2173	26,089	1.45
	20	7242	0.37	36.2	1108	26,243	
	30	5256	0.35	34.3	783	20,200	
Licomont BS 100	10	10,126	0.47	33.8	1952	28,310	1.22
	20	6992	0.38	34.1	1110	24,132	
	30	5234	0.39	33.7	786	20,310	
Rediset WMX	10	10,070	0.48	34	1928	28,859	1.31
	20	7159	0.38	34	1198	23,607	
	30	4707	0.33	30.5	667	18,305	
Ceca Base RT BIO	10	9438	0.42	31.5	1721	28,460	1.17
	20	6868	0.4	33.6	1146	22,738	
	30	4656	0.33	30.2	591	21,434	
Ceca Base RT	10	9488	0.51	31.8	1841	26,611	1.08
	20	7640	0.44	35.8	1378	22,666	
	30	5377	0.33	35	798	20,390	
